# Nasopharynx- The Secret Vault for Lost Foreign Bodies of the Upper Aerodigestive Tract

**Published:** 2016-11

**Authors:** Arijit Jotdar, Mainak Dutta, Sohag Kundu

**Affiliations:** 1 *Department of Otorhinolaryngology and Head-Neck Surgery; ICARE Institute of Medical Education and Research. Banbishnupur West Bengal, India.*; 2 *Department of Otorhinolaryngology and Head-Neck Surgery, Medical College and Hospital, Kolkata 88, College Street, Kolkata – 700073, West Bengal, India. *

**Keywords:** Foreign body, Ingested, Nasopharynx, Radiolucent

## Abstract

**Introduction:**

Foreign bodies in the upper aerodigestive tract often get lost following inappropriate attempts at removal. Children may present late with localized infection, posing a challenge to the otolaryngologists in a referral set-up in diagnosing and retrieving such foreign bodies.

**Case Report:**

A two-year-old boy presented with refractory purulent rhinorrhea and intermittent low-grade fever. The symptoms suggested rhinosinusitis; however, following a high index of suspicion, he was referred for further evaluation, with the possibility of any hidden foreign object in the upper aerodigestive tract. His soft palate appeared bulged, and his mother informed that he had ingested the cap of a plastic bottle about a month back which could not be retrieved despite several attempts by her. X-ray of soft tissue nasopharynx revealed a radiolucent shadow of a round object resulting in palatal bulging. It was eventually removed by combined endoscopic/transoral approach.

**Conclusion:**

In a child with a lost foreign body, the nasopharynx should be meticulously explored. This is less common for ingested objects compared to inhaled ones. The diagnosis becomes furthermore challenging when it is not radio-opaque. Naïve manipulations must be avoided and prompt referral should be made to the otolaryngologists for guided removal and minimizing complications.

## Introduction

The nasopharynx, the attic or vault of the human pharynx, potentially acts as the site for hidden objects in children inserting foreign bodies in their nose or mouth. This region is often overlooked in searching for lost foreign bodies in the upper aerodigestive tract, primarily because the search is chiefly directed in the cavities immediate to the facial orifices, including even the esophagus, and also because it is unusual for a foreign body, which was inhaled and lodged in the nasal cavity, to migrate and get impacted in the nasopharynx. This is further rare for ingested foreign bodies. In this brief report, we present a case of a small child with an ingested foreign body that was lost in the process of manual extraction, and was ultimately recovered from the nasopharynx.

## Case Report

A two-year-old boy was referred to the otolaryngology clinic by the pediatrics department for consultation regarding a suspected upper respiratory tract infection and rhinosinusitis and also to exclude the possibility of a hidden foreign body. History obtained from his mother revealed that he had purulent nasal discharge for about three weeks, with intermittent low grade fever since two weeks. This was associated with an occasional cough and the child had recently started refusing food. His parents consulted the local practitioner who prescribed antibiotics suspecting the condition to be a respiratory tract infection. However, although the fever subsided for the time being, the nasal purulence continued.

Upon initial examination in our out-patients’ department, the child was playful, afebrile, with no apparent sign of respiratory distress, although there was copious amount of mucopurulent discharge in the nasal cavity as evident on anterior rhinoscopy. Examination of the oral cavity and oropharynx revealed postnasal drip and slight bulging of the soft palate. 

After interrogating the child’s mother it was made known that the child had been observed puting the cap of a small plastic bottle in his mouth about a month back. Immediately thereafter he had difficulty in swallowing and pain in his throat, but there was no paroxysm of cough or dyspnea. Several attempts were made by his mother to take it out with her fingers, but without success. However, the symptoms ameliorated soon, and with the child feeding well, no further medical attention was sought and the cap was presumed to be swallowed. It was only when the sick child was referred by the general practitioner to the pediatrics department of our institute that the clinical presentation mimicking upper airway infection was considered to be caused by a probable hidden foreign body. Leading questions to his parents further strengthened the suspicion. A formal referral to the otolaryngology department was made.

**Fig1:**
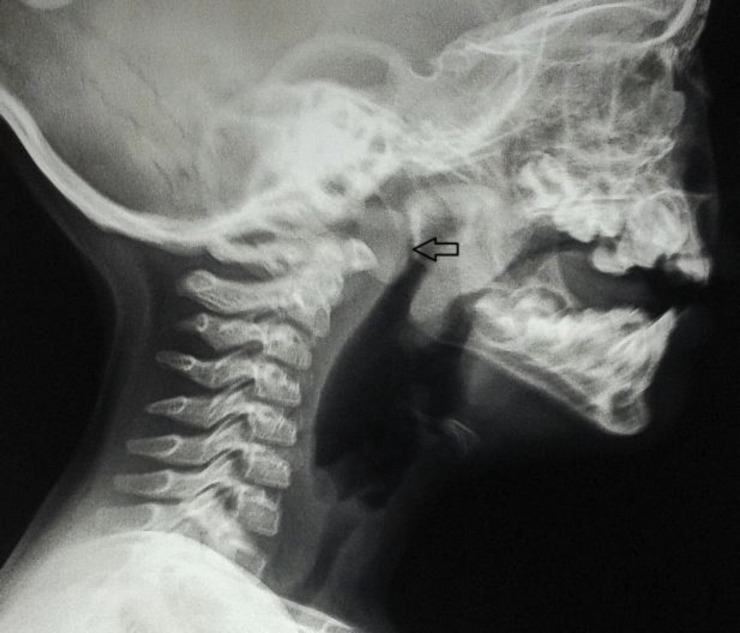
X-ray showing the round, radiolucent foreign body (arrow) in the nasopharynx abutting against the soft palate.

Considering the localized nature of the infection and mild bulging of the soft palate, we conjectured that the symptoms were more corroborative of a hidden foreign body that could have possibly been lodged in the nasopharynx. At the same time, we were also preparing for rigid esophagoscopy as the child was not feeding well. The clear lung fields on auscultation and chest x-ray and lack of suggestive history precluded us from considering a bronchial foreign body. However, a lateral view skiagram of the nasopharyngeal soft tissue revealed a radiolucent impression of some unknown round object that pushed the soft palate down (Fig.1). It was presumed to be the lost cap, and we prepared for its removal. 

**Fig 2:**
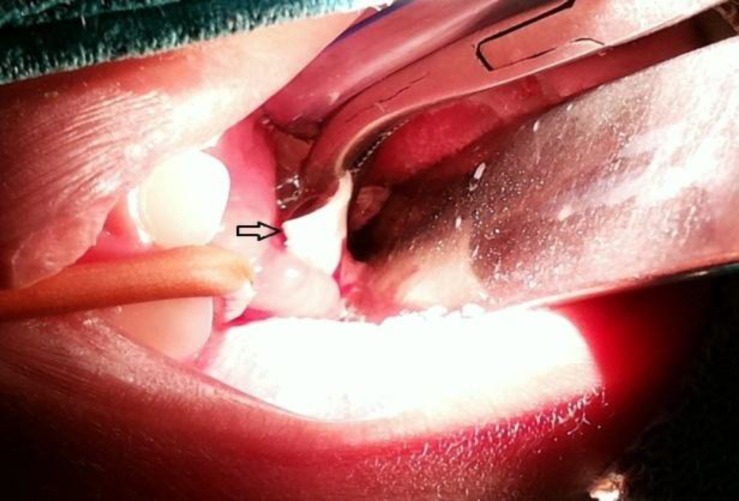
The foreign body (arrow) being retrieved from the nasopharynx per-orally.

**Fig 3:**
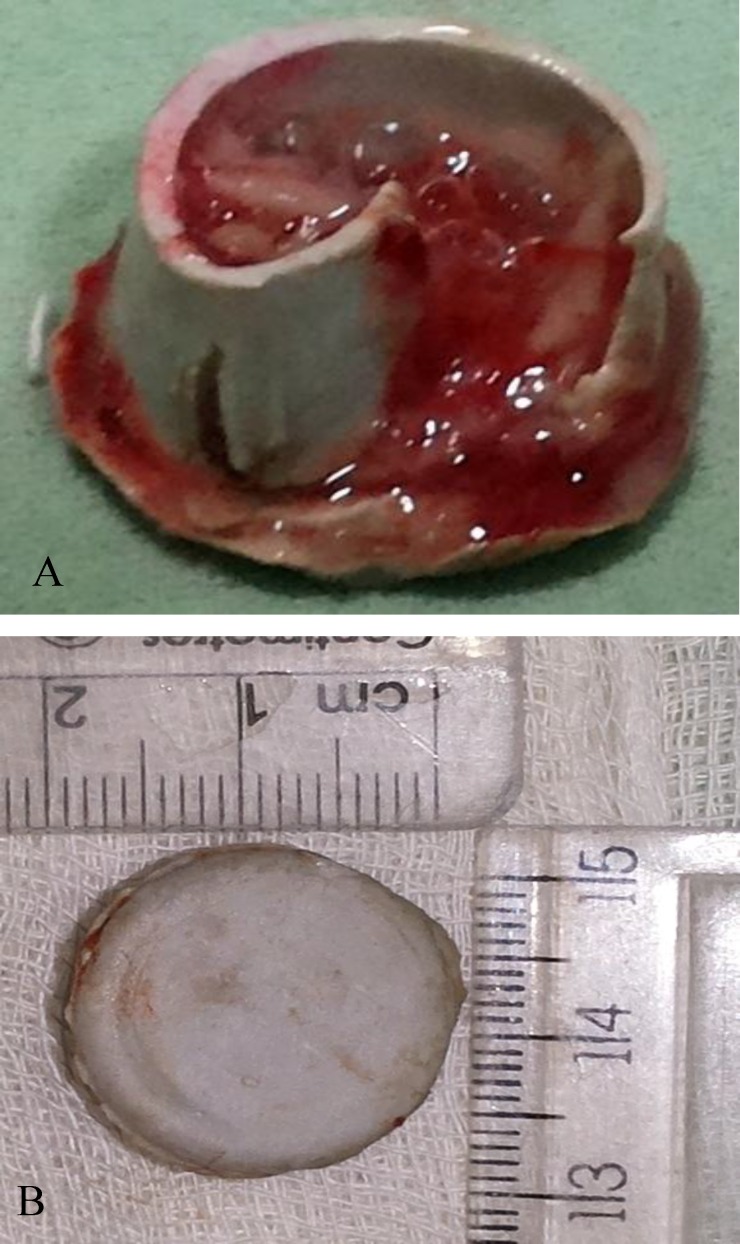
The offending agent was the rubber cap of a bottle (a,b), about 2 cm x 2 cm in dimension (b).

Following oro-tracheal intubation under general anesthesia, naso-endoscopy was performed and the foreign body was found in the nasopharynx, embedded between its posterior wall and the epipharyngeal surface of the soft palate. It could not be recognized initially as it was covered with granulations and discharge and it was too large to be delivered through the nose. A thin rubber catheter was passed through the left nasal cavity under endoscopic guidance and the other end was taken out from the oral cavity, retracting the soft palate anteriorly. The foreign body was now dislodged from its niche and delivered per-orally with the help of Tiley’s forceps (Fig.2). It was a soft rubber cap measuring about 2 cm x 2 cm (Fig.3a,b). 

Note that the cap is coated with granulations and secretions (Fig.3a).

The child was put on antibiotics for seven days along with regular nasal cleansing with isotonic normal saline. He recovered well without complications and was asymptomatic during his three month follow-up. 

The present report has been prepared after obtaining permission from the Institutional Ethical Committee and informed consent from the child’s parents.

## Discussion

It is not surprising for children to insert objects in their mouth or nose as they are inquisitive of their natural body orifices (1). Many times they are removed by their parents, but not always, and in those instances they are often considered lost if the child remains asymptomatic. However, in occasional instances these foreign bodies are found in the nasopharynx, which is not usual (2). This is because the nasopharynx is an area that is often overlooked and is a potential site for impaction of foreign objects that could not be retrieved and, with time, forgotten. Most of them are inhaled. We have recently reported a lost metallic ring that was inserted in the nasal cavity by a three-year-old boy and was subsequently found anchored in the thick adenoid tissue (3). However, an ingested foreign body getting lodged in the nasopharynx is even more rare with only a handful of reported cases (4). These mostly result from naïve attempts at removal by the parents, primary-care physicians (5), or quacks. Rarely, vigorous cough or vomiting can push the ingested agent into the nasopharynx or when it is put into the mouth in the head-down position (5). From the available clinical history in this child, it appears that following the blind digital manipulations during the attempted removal the cap migrated and subsequently lodged itself in the nasopharynx. 

Diagnosis of nasopharyngeal foreign body requires a low threshold of clinical suspicion. Skiagram of the nasopharyngeal soft-tissue might reveal a radio-opaque foreign body. But the problem arises for radiolucent objects in which case the diagnosis becomes challenging. The child may suffer from features of localized infection like nasal purulence and obstruction and in many cases the symptoms appear late (6). A retained nasopharyngeal foreign body also carries the risk of getting dislodged resulting in swallowing difficulty and potentially fatal respiratory distress (4,7). The management therefore entails emergency naso-endoscopic removal and occasionally removal through the oral cavity under endoscopic guidance in difficult cases or when the foreign body is too large. 

Evidently, foreign bodies get lodged in the nasopharynx chiefly due to inexperienced and unsuccessful attempts at removal. There is a belief that the child has swallowed it, especially when he/she remains asymptomatic. Although this conjecture is not always wrong, a proportion of this group often has the object in their nasopharynx, which acts as a hidden pocket. The foreign body remains there, undiagnosed, resulting in a delay in diagnosis and a late presentation with potential complications. Therefore, it must be stressed that children with a history of foreign body insertion should be promptly referred to otolaryngologists. The clinical situation is ostensibly simple but potentially hazardous when attempts at removal fail and needs to be treated as an emergency requiring specialized surgical expertise. The nasopharynx should be the chief area where the search should be directed and should always be considered as an area of concern in the context of a lost foreign body (7). The difficulty in diagnosis is further complicated when the foreign object is not radio-opaque and imaging is not helpful. The bottle cap in this child was originally radio-transparent, but prolonged stay in the nasopharynx presumably allowed it to get coated with granulations and secretions turning it into a translucent entity with preserved contours. We were fortunate to be able to diagnose it by a simple x-ray, focusing on the soft-tissue nasopharynx, and perform a directed surgical approach. Otherwise, as it happens in most cases, the exploration would have been more difficult and traumatic.

## Conclusion

The nasopharynx acts as a secret blind area where lost foreign bodies are often harbored. Such situations are more common for inhaled foreign bodies and relatively rare when they are ingested. In either case, lodgment of foreign bodies mostly occurs following inexperienced attempts at removal that often invite potentially preventable complications. Children with a suggestive history and clinical features of ingestion/inhalation of foreign bodies should be promptly referred to otolaryngologists for expert management. From the otolaryngologists’ perspective, the nasopharynx should always be checked while searching for lost, displaced foreign objects in the upper aerodigestive tract.
